# The effect of norethisterone acetate on the uterine telocytes, immune cells and progesterone receptors in albino rats

**DOI:** 10.1038/s41598-025-92354-5

**Published:** 2025-03-15

**Authors:** Mahmoud Abd-Elkareem, Sulaiman Mohammed Alnasser, Alotaibi Meshal, Mohamed H. Kotob, Ayman S. Amer, Raghda Ismail Abdullah, Ahmed U. Ali

**Affiliations:** 1 Department of Cell and Tissues, Faculty of Veterinary Medicine, Assiut University, Assiut, 71526 Egypt; 2 Department of Pharmacology and Toxicology, College of Pharmacy, Qassim University, 52571 Buraydah, Saudi Arabia; 3 Pharmacy Practice, College of Pharmacy, University of Hafr Albatin, Hafr Albatin,, Saudi Arabia; 4 Division of Pharmacology and Toxicology, Department of Pharmaceutical Sciences, University of Vienna, 1090 Vienna, Austria; 5 Department of Pathology, Faculty of Veterinary Medicine, Assiut University, Assiut, 71526 Egypt; 6 Department of Human Anatomy and Embryology, Faculty of Medicine, Assiut University, Assiut, 71526 Egypt; 7 Department of Anatomy, Histology and Embryology, Faculty of Veterinary Medicine, New Valley University, El Kharga, Egypt; 8 Faculty of Pharmacy, Assiut University, Assiut, Egypt

**Keywords:** Norethisterone acetate, Albino rat, Immune cells, Telocytes, Progesterone receptors, Eosinophils, Mast cells, Uterus, Cell biology, Immunology, Anatomy, Endocrinology

## Abstract

This study is the first attempt to examine the effects of NETA on immune cells and telocytes. The results of this study form an important knowledge base for the development of new information on the mechanism of contraceptive action of NETA in the uterus. Norethisterone acetate (NETA) is a synthetic progestogen medication commonly utilized in birth control pills, menopausal hormone therapy, and for curing abnormal uterine bleeding and endometriosis. Furthermore NETA has many beneficial uses in veterinary medicine as control and synchronization of estrous cycle. The impact of NETA on the endometrial stromal cells (ESCs), telocytes, and uterine immune cells is not well understood. Therefore, this study focuses on assessing changes in uterine immune cells, ESCs, and telocytes following exposure to NETA in albino rats. To achieve this objective, fourteen adult female albino rats were randomly divided into two groups: a control group and an NETA-treated group. Rats in the control group received daily pelleted food, water, and were oral administered of 2 ml distilled water. In contrast, rats in the NETA-treated group received daily pelleted food, water, and were orally administered 20 µg of NETA dissolved in 2 ml distilled water. The experiment spanned three weeks. The findings of this study revealed that NETA usage increases the infiltration and activity of immune cells (eosinophils, neutrophils, macrophages, lymphocytes, and mast cells). Furthermore, it enhances the vesicular activity of uterine telocytes and their communication with various immune cells. NETA also influences decidualization and the immunoexpression of progesterone receptors in uterine epithelial and immune cells. This study concludes that the primary mechanism by which NETA controls pregnancy is through decidual (pregnancy-like) effects or improper decidualization, which inhibits fertilization and implantation respectively. Our research provides evidence of the contraceptive mechanism of NETA from an immunological perspective in an animal model.

## Introduction

A vital part of preventative healthcare is getting a contraceptive that is safe, effective, and reasonably priced. Progestin-only contraception provides a safe and effective control of pregnancy. The contraceptive activity of synthetic progestins is mediated through three basic mechanisms: (a) An anti-gonadotrophic action leading to the prevention of ovulation; (b) Alter the cervical mucus characteristics that inhibit sperm penetration and (c) Desynchronization of the uterine picture necessary for implantation^1^. Norethisterone (Norethindrone) acetate (NETA) is the first orally active progestogen synthesized, and is referred to as a first generation progestogen. NETA are widely used as female contraceptive agents, in hormone replacement therapy and for the treatment of many gynecological disorders^2^. Moreover, Norethisterone reduces vaginal bleeding from birth control pills containing only progesterone^3^.

Many oral contraceptives were used in USA and Europe to control Wildlife mammals and birds^4^. Moreover, in wildlife, contraception may be used as a management technique for some illnesses, particularly those that are contracted during parturition or are spread via sexual contact. One important infectious disease that is prevalent in wild populations and may be controlled by immunocontraception is brucellosis^5^. Contraceptive drugs such as synthetic progestin can be used to control and prevent estrus in pets (dogs and cats). This often provides rapid suppression of undesired female behavior during estrus and overcome the risk of surgical intervention^6^. Also many hormonal contraceptive drugs were used to control and synchronize the estrous cycle in different domestic animals such as cow^7,8^, sheep and goats^9^, she camel^10^ and mares^11^.

Norethisterone changes the normal structure and physiology of the female genital tract, thereby inhibiting conception in the albino rat^12,13^. Ovarian follicles are negatively impacted by NETA, exhibiting atresia at all stages and a decrease in quantity^14^. Since Norethisterone do not exert immunosuppressive properties at physiological concentrations, these progestins are considered as alternative contraceptives for women at high risk of infection^2^. In spite of progestins act primarily via the progesterone receptor (PR); they exert various side effects due to their diverse affinities to other members of the steroid receptor family^2,15^. As it was suggested that progestins exert their effect on immune mechanisms primarily via the glucocorticoid receptors (GR)^2,15,16^. GR is a well-established regulator of immune system^2^.

Some progestins and combined oral contraceptives suppresses the function of T cells and dendritic cells leading to altered immune environment in the female reproductive tract^2,17^.

Regretfully, despite significant study efforts, there remains an inadequate understanding regarding the impact of NETA on uterine immune cells and telocytes, necessitating a more profound comprehension of the underlying mechanisms. So, the purpose of the present study is to give more information on the effects of NETA on the uterine immune cells, ESCs and telocytes and the relationships between them. In addition to elucidate the mechanism involved in NETA contraception.

## Materials and methods

### Source of the animals

Female albino rats were sourced from the animal facility within the Faculty of Veterinary Medicine at Assiut University, Assiut, Egypt. These rats were provided with a diet of commercial pelleted food and housed in metal cages with unrestricted access to water and food. The duration of the experiment spanned three weeks. The experimental protocol received approval from the Local Ethical Committee and the Institutional Review Board of the Molecular Biology Research and Studies Institute at Assiut University (IORG0010947-22-2024-0010), and was carried out in accordance with relevant guidelines and regulations. This research was done in compliance with the ARRIVE guidelines and regulations (https://arriveguidelines.org/). All national and institutional guidelines for animal care and use have been followed throughout the study procedures.

### **Drug**

Norethisterone acetate was acquired from Chemical Industries Development (CID) Company in Giza, Egypt.

### Experimental design

A total of 14 non-pregnant female albino mature rats, with an average body weight ranging from 150 to 180 g and an average age of 2 to 3 months, were randomly divided into two groups following acclimatization. Each group comprised seven animals.

In the control group, each mature rat was orally administered 2 ml of distilled water daily. The rats were also provided with water and a commercially pelleted diet for duration of three weeks.

In the NETA treated group, each rat was received a daily oral dose of 20 µg of NETA dissolved in 2 ml of distilled water. Similar to the control group, the rats in this group were supplied with water and a commercially pelleted diet for a period of three weeks.

Due to the potency of the drug and the impracticality of weighing or adjusting the dose individually for each animal, NETA was diluted with lactose^18^. After geometrically diluting 4 mg of NETA with 996 mg of lactose, 5 mg of the mixture (which contains 20 µg of NETA) was dissolved in 2 ml of distilled water^13^.

### Histological preparation

At the end of the experiment, rats were euthanized by cervical dislocation and uteri were dissected and fixed in 10% neutral buffered formalin.

The fixed materials were dehydrated in ascending grades of alcohol, cleared in methyl benzoate, and then embedded in paraplast. Paraffin sections of 5 μm in thickness were cut and stained with Hematoxylin and Eosin for general histological examination of the uterus^19^.

### Immunohistochemical detection of mast cells and progesterone receptor

For detection of mast cell, we used Mast Cell Tryptase (3G3) Monoclonal Antibody (Bioss antibodies) and for detection of progesterone receptors we used Progesterone Receptor Polyclonal Antibody (Bioss antibodies). Additionally, the procedure included the utilization of the Poly Q stain 2-step detection system, goat anti-mouse/rabbit HRP, peroxidase quench, and DAB kit from Quartett, Germany. The immunohistochemical protocol adhered to the instructions provided by the respective companies, as well as following our previous work^20^.

### Semithin sections and transmission electron microscopic preparations

Small pieces of uteri were immersed in a solution of 2.5% glutaraldehyde in phosphate buffer (pH 7.2) for a period of 24 h. Following fixation, the specimens were washed in 0.1 M phosphate buffer and subsequently post-fixed in 1% osmium tetraoxide. Dehydration was carried out using ascending concentrations of alcohol, and the specimens were then embedded in araldite resin. Staining of semithin sections, with a thickness of 1 μm, was performed using 1% toluidine blue. Ultrathin sections were produced using a Reichert ultra-microtome, followed by staining with uranyl acetate and lead citrate. The examination and capturing of electron micrographs from the ultrathin sections were conducted using a Jeol Jem 1200 EX Transmission Electron Microscope at the Electron Microscope Center of Assiut University^21^.

For the evaluation of paraffin sections and semi-thin sections, an OLYMPUS BX51 microscope was utilized, and images were captured using an OLYMPUS DP72 camera integrated into the microscope.

### Counting of tryptase positive mast cells per microscopic filed

The number of tryptase positive mast cells was counting per microscopic filed.

### Statistical analysis

The data were presented as mean values ± stander error of means (S.E.) The data were subjected to statistical analysis using SPSS 16 and the independent-samples t-test. The value was considered significance at (*P* < 0.05).

## Results

**Fig. 1:**
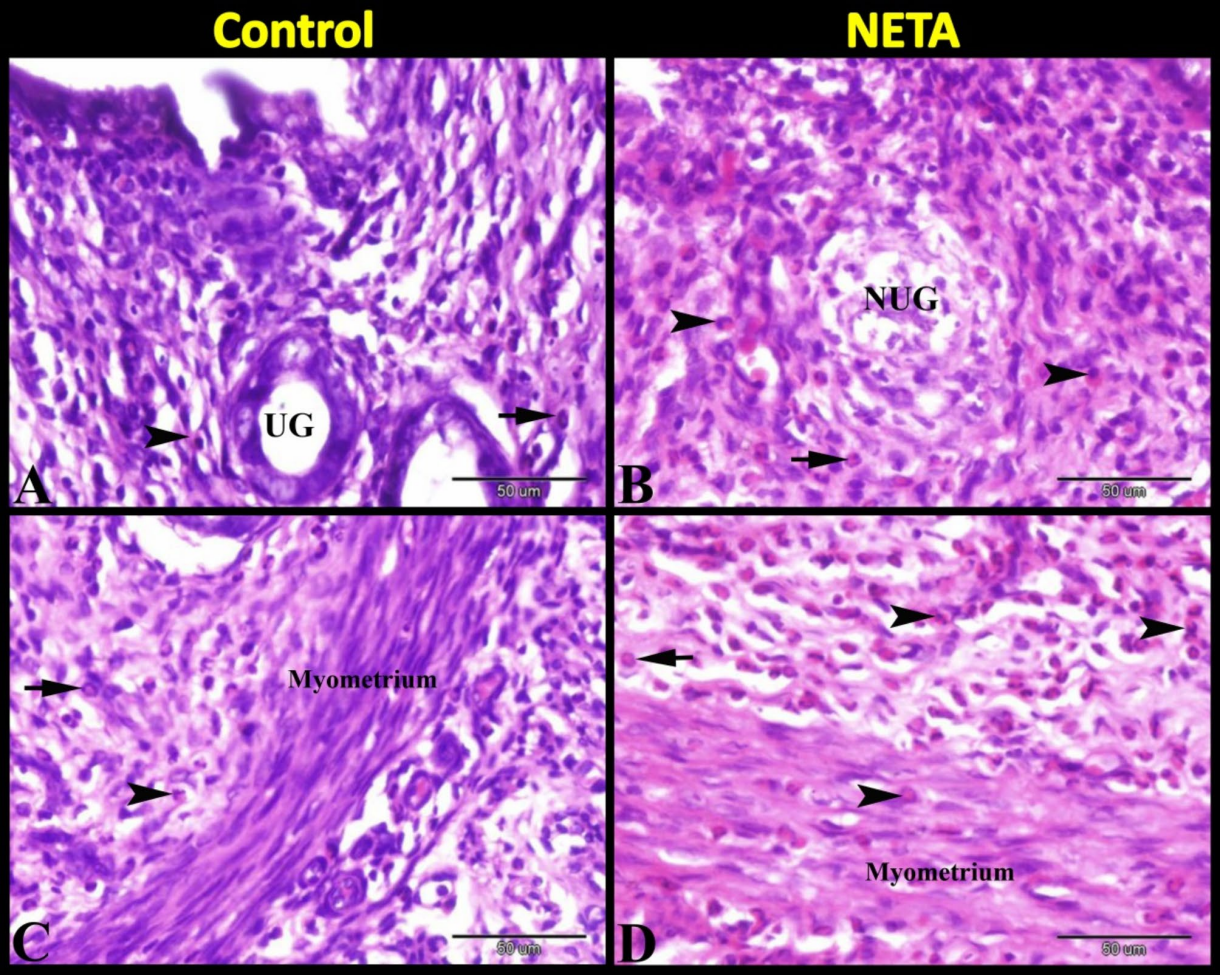
Photomicrograph of paraffin sections in rat uterus stained with Hx & E showing the effect of NETA on uterine immune cells. (**A**) Control group showing uterine endometrium with uterine glands and few polymorph leucocytes; neutrophils (arrow) and eosinophils (arrowheads). (**B**) NETA treated group showing uterine endometrium with necrotic uterine gland (NUG) and infiltration of abundant polymorph leucocytes; neutrophils (arrow) and eosinophils (arrowheads). (**C**) Control group showing the deep layer of endometrium and uterine myometrium with few polymorph leucocytes; neutrophils (arrow) and eosinophils (arrowheads). (**D**) NETA treated group showing the deep layer of endometrium and uterine myometrium with infiltration of abundant polymorph leucocytes; neutrophils (arrow) and eosinophils (arrowheads). Scale bar A–D = 50 μm.

**Fig. 2:**
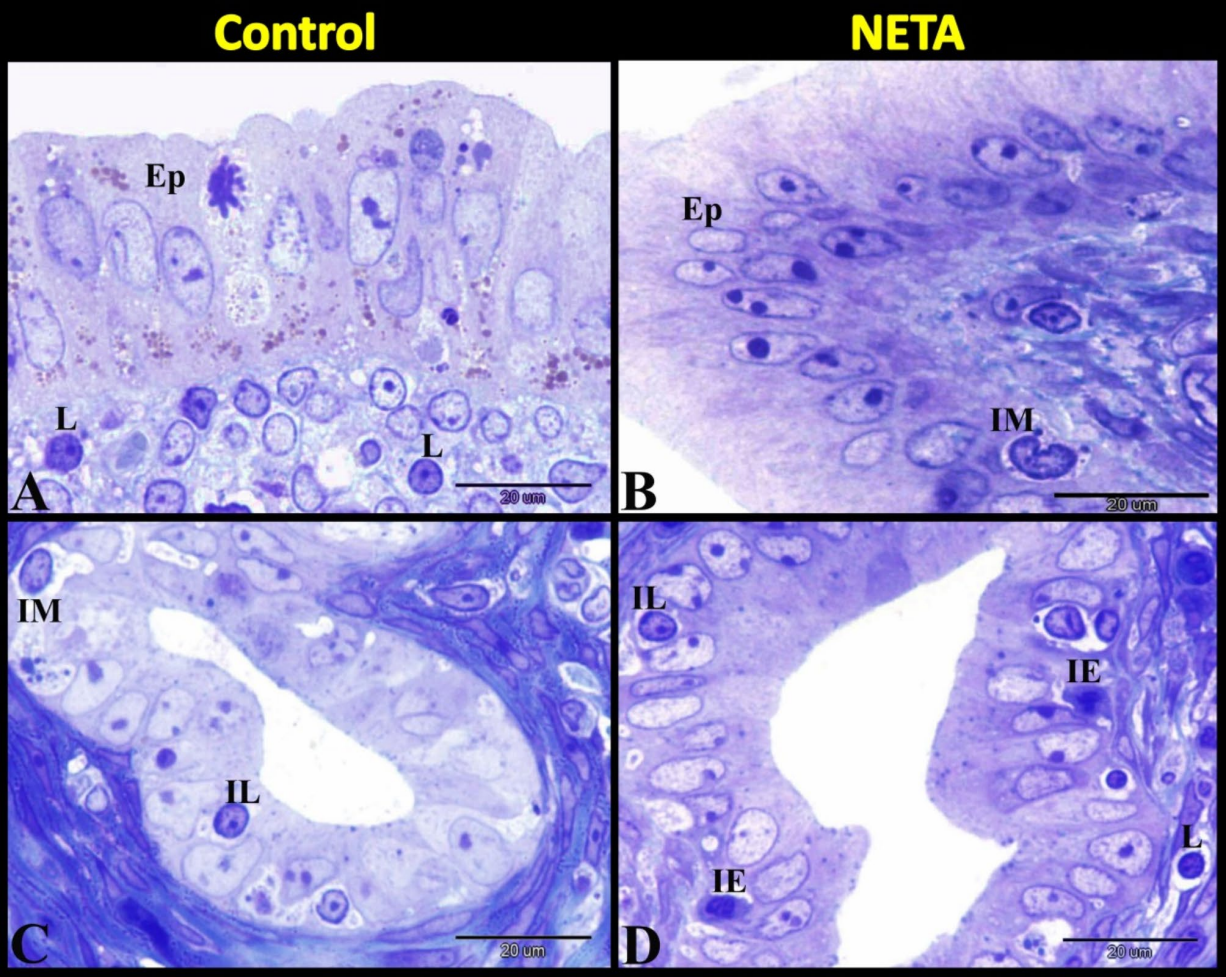
Photomicrograph of semithin sections in rat uterus stained with toluidine blue showing the effect of NETA on uterine immune cells. (**A**) Control group showing uterine epithelium (Ep) with sub-epithelial lymphocytes (L). (**B**) NETA treated group showing uterine epithelium (Ep) with intraepithelial macrophage (IM). (**C**) Control group showing the uterine gland with intraepithelial lymphocytes (IL) and macrophage (IM). (**D**) NETA treated group showing the uterine gland with intraepithelial lymphocytes (IL) and eosinophils (IE). Scale bar A–D = 20 μm.

Microscopic examination of the rat uterus after oral administration of NETA revealed that there were abundant infiltrations of polymorph leucocytes; neutrophils and eosinophils in superficial (around uterine glands) and deep layers of endometrium and in myometrium in NETA treated group compared to control (Fig. 1A-D). In control group we observed sub-epithelial and intraepithelial lymphocytes and macrophages. Whereas in NETA treated group we observed the uterine glands with intraepithelial lymphocytes and eosinophils while the endometriual epithelium with intraepithelial macrophages (Fig. 2A–D).

**Fig. 3:**
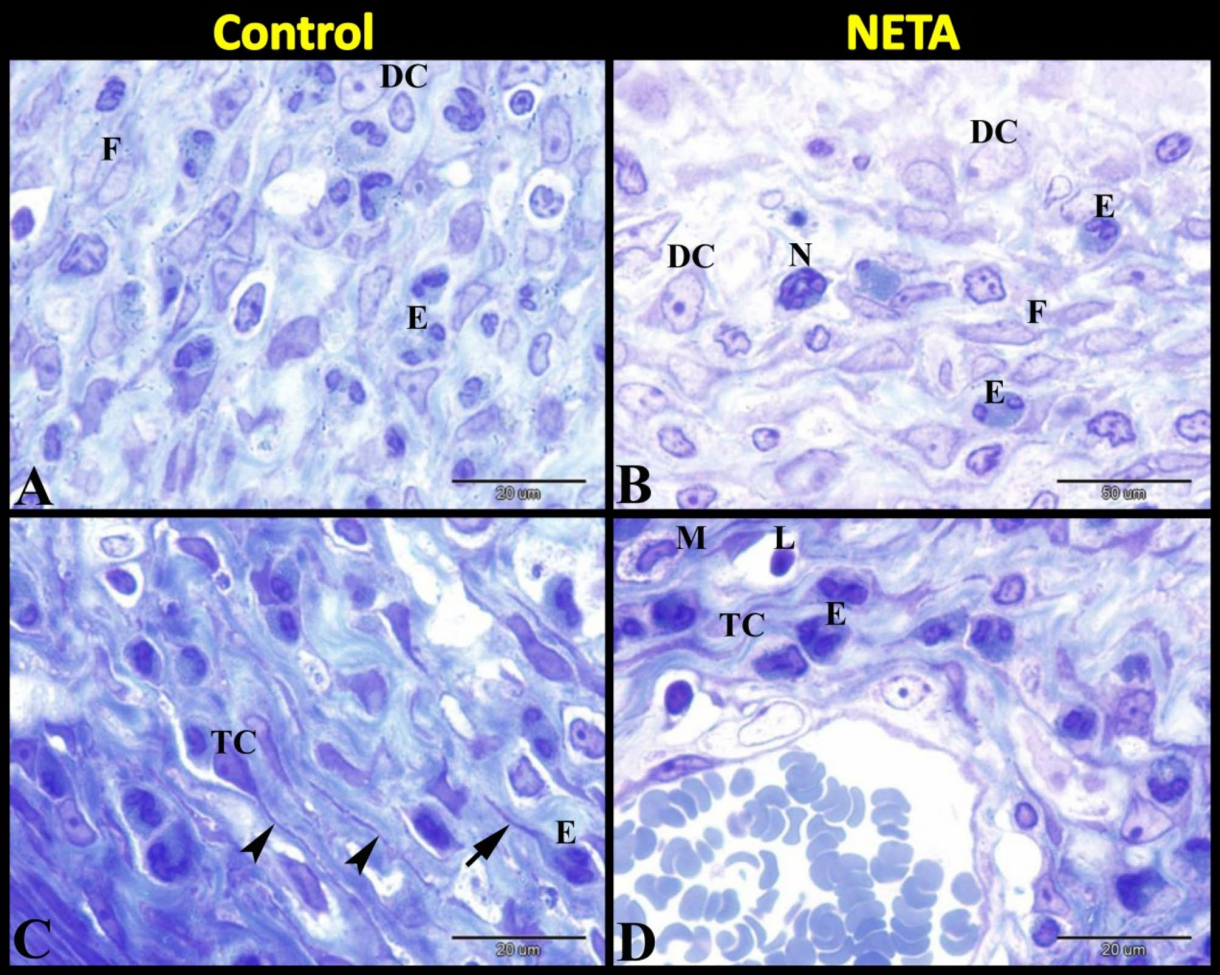
Photomicrograph of semithin sections in rat uterus stained with toluidine blue showing the effect of NETA on the endometrial cells. (**A**) Control group showing the endometrium contained fibroblasts (F), eosinophils (E) and few decidual cells (DC). (**B**) NETA treated group showing the endometrium contained fibroblasts (F), eosinophils (E), neutrophils (N) and numerous decidual cells (DC). (**C**) Control group showing the endometrium contained many telocytes (TC) with its characteristic long telopodes (arrowheads). Note some telocytes differentiated into eosinophil (E) and still contained telopodes (arrow). (**D**) NETA treated group showing the endometrium contained many telocytes (TC), eosinophils (E) and macrophage (M). Scale bar A–D = 20 μm.

Our findings showed that the endometrial stroma in control group contained fibroblasts, eosinophils and few decidual cells. Also, it contained many telocytes with its characteristic long telopodes. We demonstrated that some telocytes were differentiated into eosinophil and still contained telopodes. But in NETA treated group the endometrial stroma contained fibroblasts, eosinophils, neutrophils, telocytes, macrophages and numerous decidual cells (Fig. 3A–D).

**Fig. 4:**
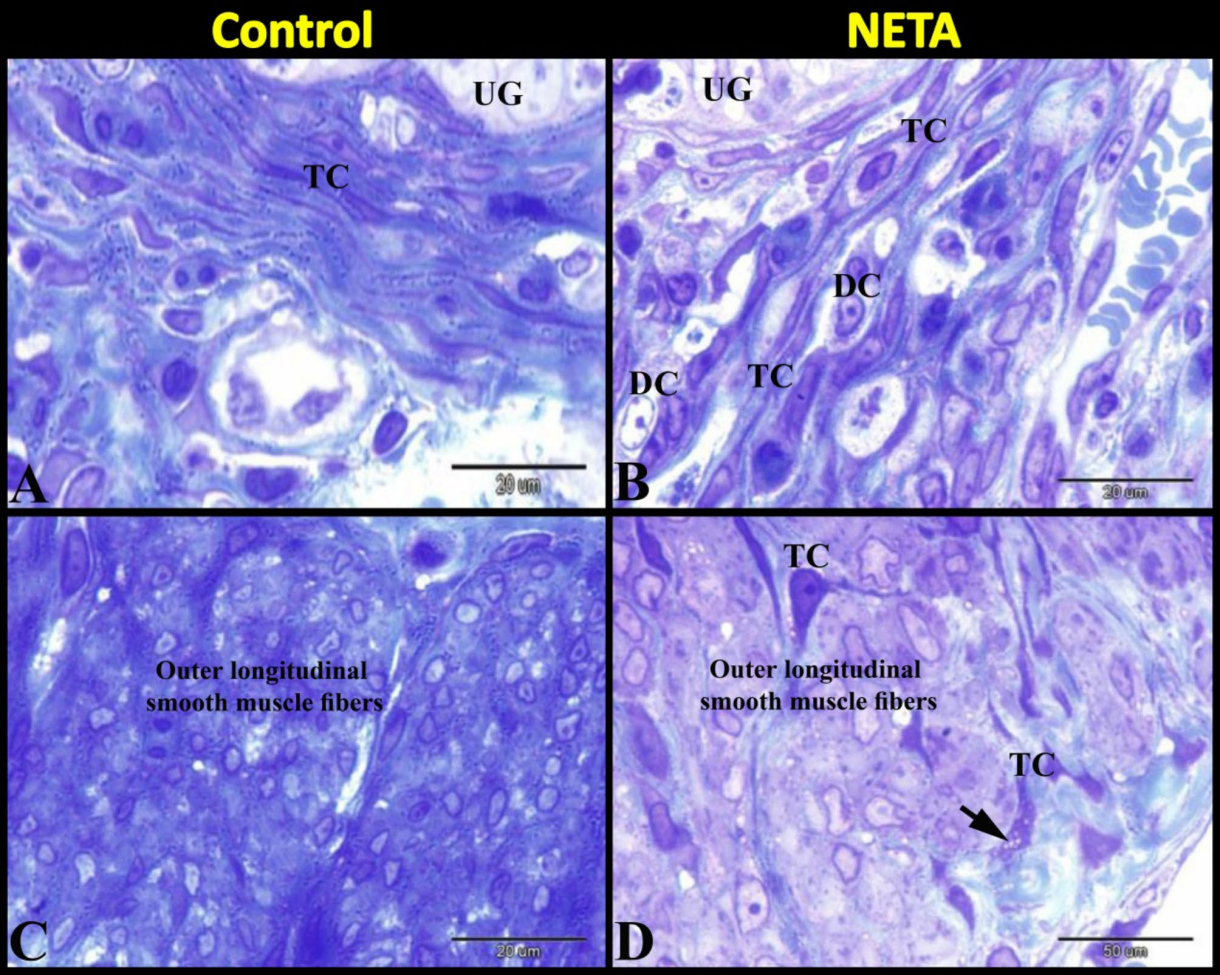
Photomicrograph of semithin sections in rat uterus stained with toluidine blue showing the effect of NETA on the telocytes. (**A**) Control group showing the endometrium contained many telocytes (TC) around the uterine glands (UG). (**B**) NETA treated group showing the endometrium contained many telocytes (TC) around the uterine glands (UG) and numerous decidual cells (DC) in between them. (**C**) Control group showing the outer longitudinal smooth muscle layer of myometrium contained few or no telocytes (TC). (**D**) NETA treated group showing the outer longitudinal smooth muscle layer of myometrium contained many telocytes (TC) with vesicular cytoplasm (arrow). Scale bar A–D = 20 μm.

Herein we found that in the control group many telocytes were demonstrated around the uterine glands (Fig. 4A). In NETA treated group we also observed many telocytes around the uterine glands but with numerous decidual cells in between them (Fig. 4B). In the control group the outer longitudinal smooth muscle layer of myometrium contained few or no telocytes (Fig. 4C). Whereas in the NETA treated group the outer longitudinal smooth muscle layer of myometrium contained many telocytes with vesicular cytoplasm (Fig. 4D).

**Fig. 5:**
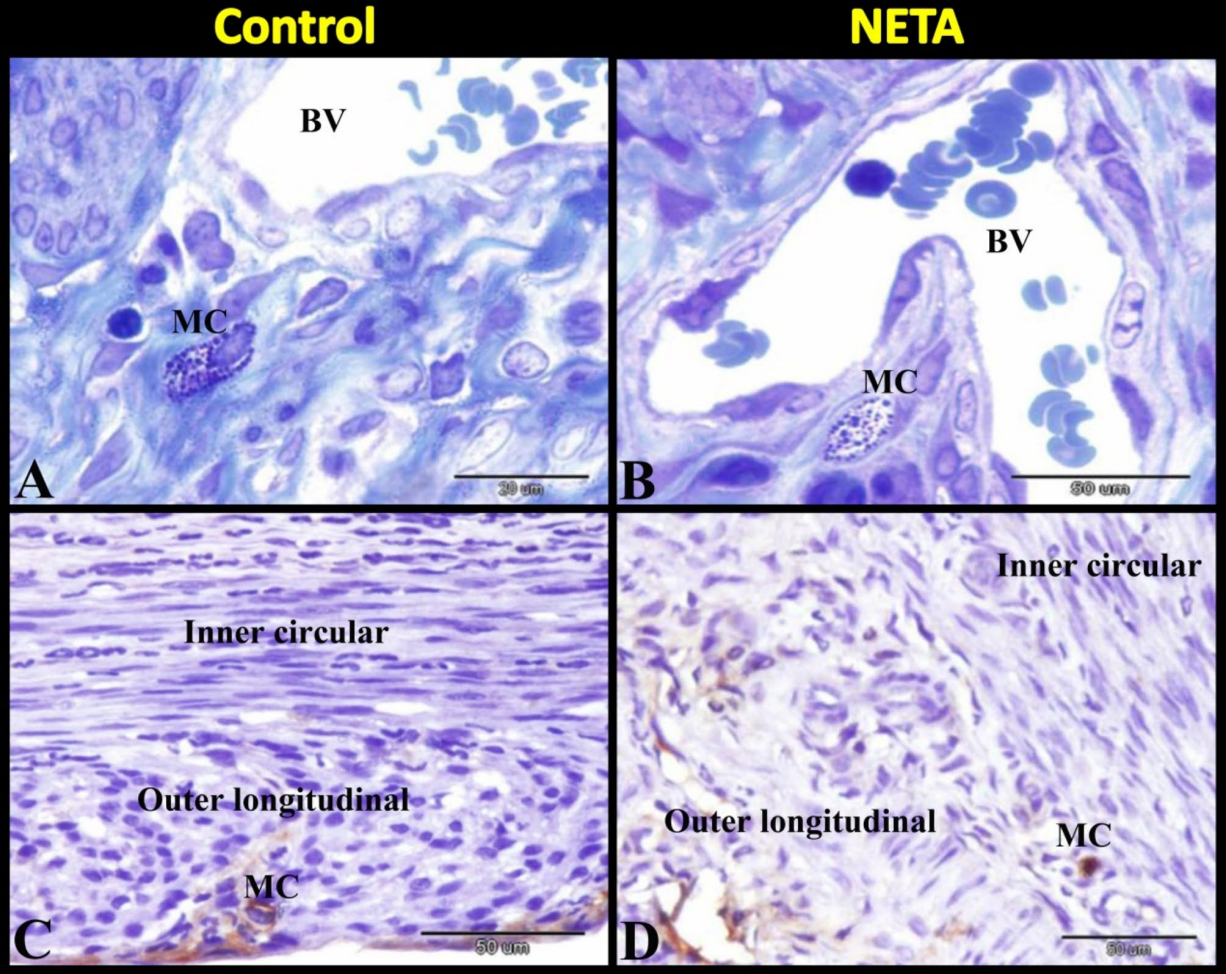
Photomicrograph of semithin sections stained with toluidine blue (**A** & **B**) and mast cell tryptase immunostaining (**C** & **D**) in rat uterus showing the effect of NETA on the mast cells. (**A**) Control group showing resting mast cell (MC) with its characteristic metachromatic granules around blood vessel (BV) in the myometrium. (**B**) NETA treated group showing activated mast cell (MC) with somewhat few metachromatic granules around blood vessel (BV) in the myometrium. (**C**) Control group showing tryptase positive immunostaining mast cell (MC) in the outer longitudinal smooth muscle layer of myometrium. (**D**) NETA treated group showing tryptase positive immunostaining mast cell (MC) in between the inner circular and outer longitudinal smooth muscle layer of myometrium. Scale bar A & B = 20 μm, C & D = 50 μm.

**Fig. 6:**
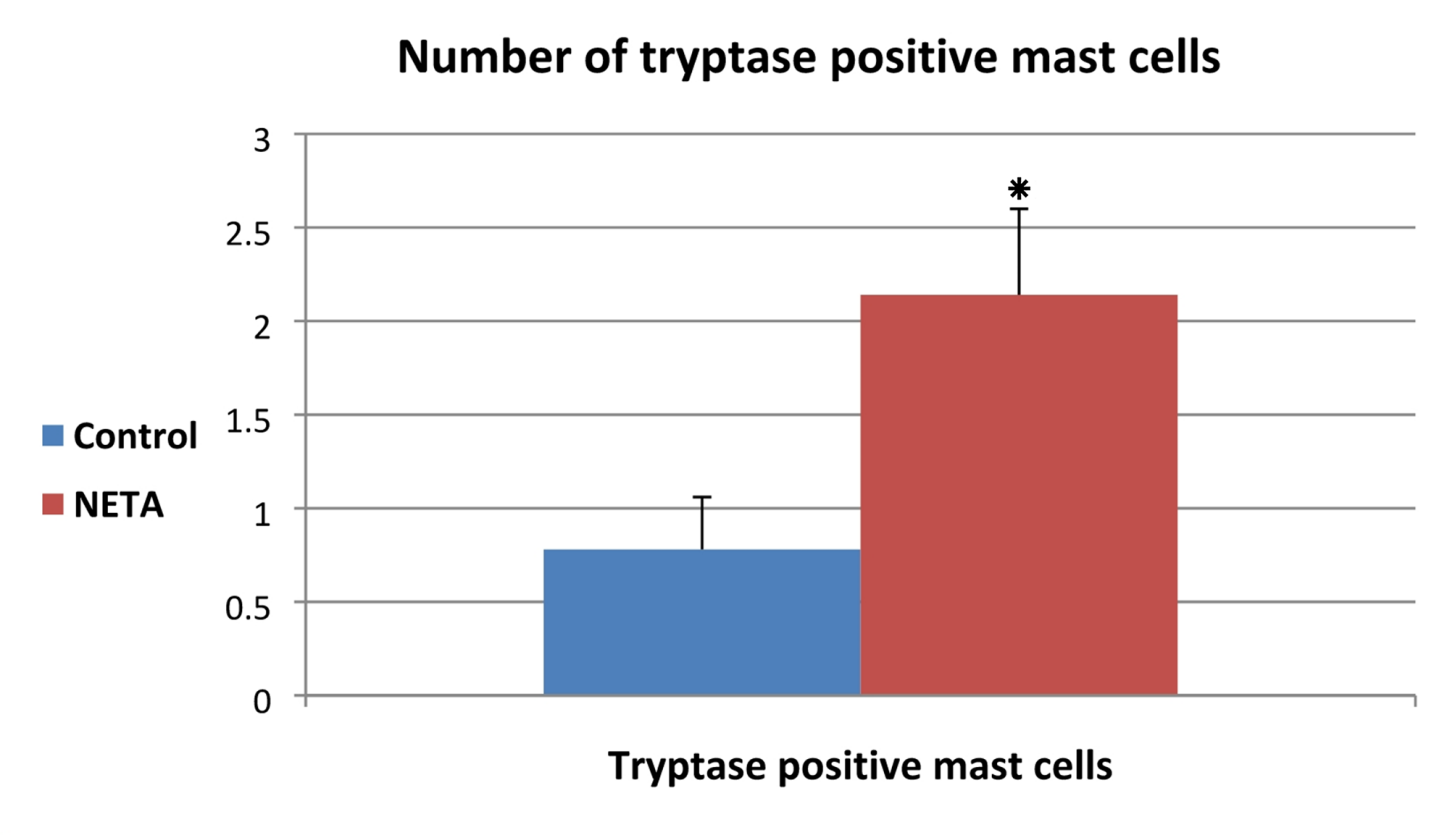
Showing the number of tryptase positive mast per microscopic filed. * = Statistically significance difference (*P* < 0.05) between NETA and control group.

Our results revealed that resting mast cell with its characteristic metachromatic granules were observed around blood vessel in the myometrium of the control group (Fig. 5A). While NETA treated group showed activated mast cell with somewhat few metachromatic granules around blood vessel in the myometrium (Fig. 5B). Tryptase positive immunostaining mast cell was demonstrated in the outer longitudinal smooth muscle layer of myometrium in control group (Fig. 5C). However NETA treated group showed tryptase positive immunostaining mast cell in between the inner circular and outer longitudinal smooth muscle layer of myometrium (Fig. 5D). Interestingly, we found that the number of tryptase positive mast cells was significantly increased in NETA treated group compared to control (Table 1; Fig. 6).

**Table 1:** Showing the number of tryptase positive mast cells per microscopic filed.

Groups	Number of tryptase positive mast cells
Control	0.78 ^a^ ± 0.28
NETA	2.14 ^b^ ± 0.46

Values (Means ± SE) with different superscripts (a and b) in the same column are significantly different (*P* < 0.05) between control and NETA groups.

**Fig. 7:**
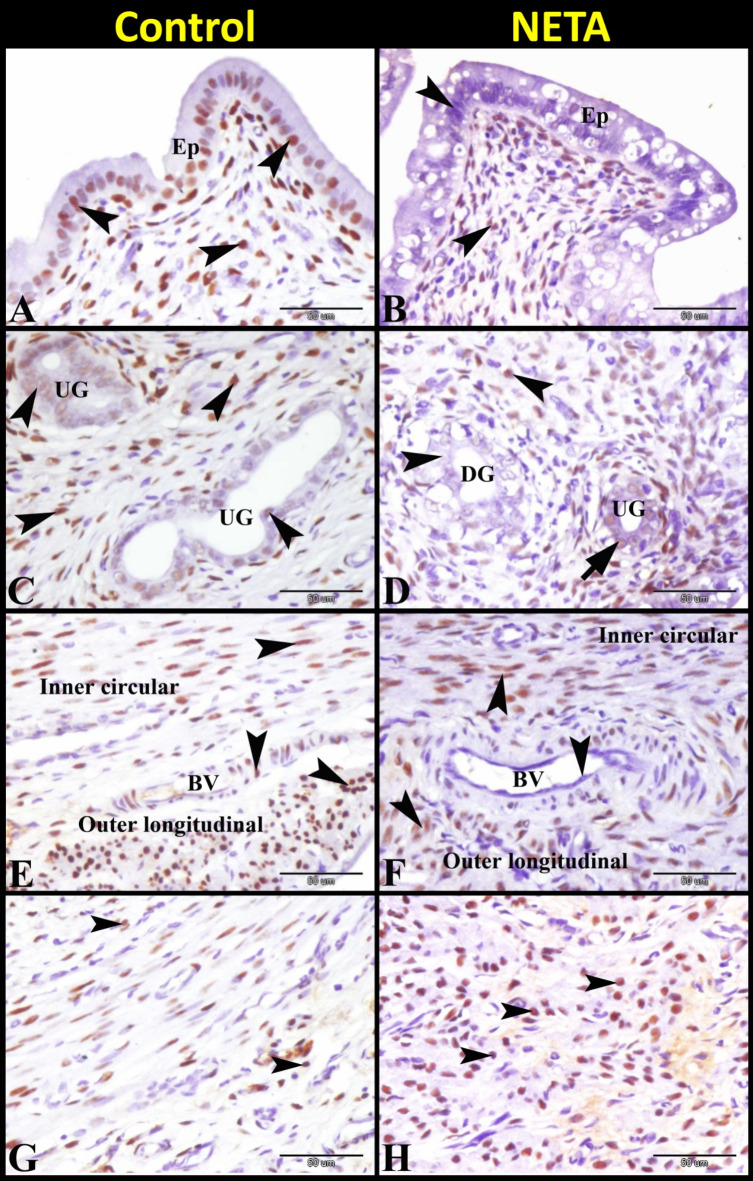
Photomicrograph of paraffin sections in the rat uterus showing the effect of NETA on the PR immunostaining. (**A**) Control group showing strong PR immunostaining (arrowheads) in the epithelium (Ep) and in the underlying stroma cells (arrowheads) of the endometrium. (**B**) NETA treated group showing negative PR immunostaining (arrowheads) in the epithelium (Ep) and moderate PR immunostaining in the underlying stroma cells (arrowheads) of the endometrium. (**C**) Control group showing moderate PR immunostaining in the glandular epithelium (arrowheads) and strong PR immunostaining in the stroma cells (arrowheads) surrounded the uterine glands (UG). (**D**) NETA treated group showing negative PR immunostaining in the glandular epithelium (arrowheads) of degenerated uterine glands (DG), moderate PR immunostaining in the glandular epithelium (arrow) of healthy uterine glands (UG) and moderate PR immunostaining in the stroma cells (arrowheads) surrounded the uterine glands (UG). (**E**) Control group showing strong PR immunostaining (arrowheads) in the inner circular and outer longitudinal smooth muscle fibers and endothelium of blood vessels (BV) in the myometrium. (**F**) NETA treated group showing strong PR immunostaining (arrowheads) in the inner circular and outer longitudinal smooth muscle fibers and negative PR immunostaining in the endothelium (arrowheads) of the blood vessels (BV) in the myometrium. (**G**) Control group showing moderate PR immunostaining (arrowheads) in the leucocytes (**H**) NETA treated group showing strong PR immunostaining (arrowheads) in the leucocytes. Scale bar A–F = 50 μm.

Immunoexpression of PR in rat uterus revealed that the uterus in the control group showed strong PR immunostaining in the epithelium and in the underlying ESCs of the endometrium. Also it showed moderate PR immunostaining in the glandular epithelium and strong PR immunostaining in the inner circular and outer longitudinal smooth muscle fibers and endothelium of blood vessels in the myometrium. While the uterus in NETA treated group showed negative PR immunostaining in the surface epithelium and glandular epithelium of degenerated uterine glands and moderate PR immunostaining in the underlying ESCs of the endometrium and glandular epithelium of healthy uterine glands. Also it showed strong PR immunostaining in the inner circular and outer longitudinal smooth muscle fibers and negative PR immunostaining in the endothelium of the blood vessels in the myometrium (Fig. 7A–F). In addition, we reported presence of moderate PR immunostaining leucocytes in control group (Fig. 7G) whereas abundant strong PR immunostaining leucocytes were observed in NETA treated group (Fig. 7H).

**Fig. 8:**
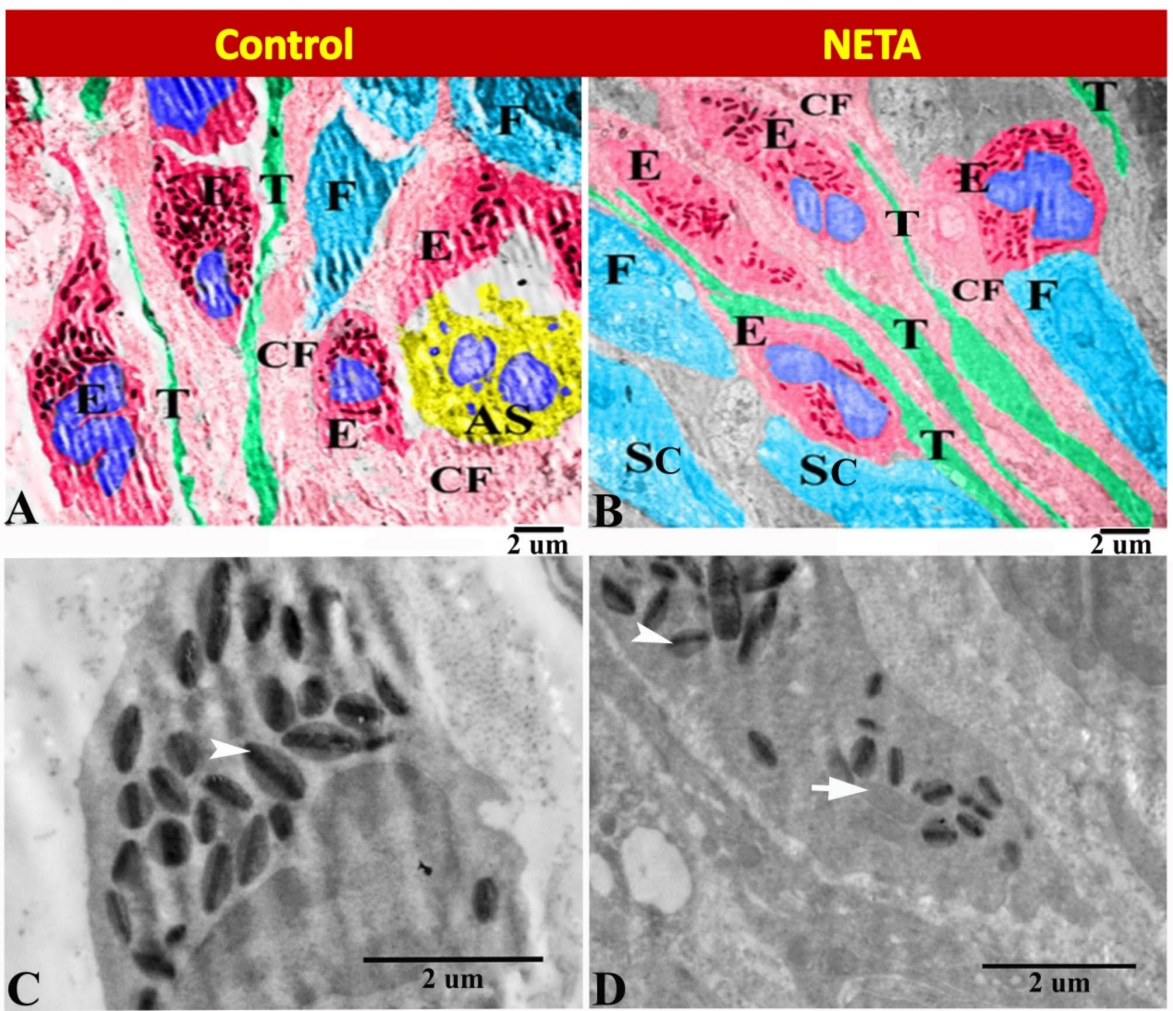
Transmission electron micrograph in the rat endometrium showing the effect of NETA on the endometrial stroma. (**A**) Control group showing the endometrium contained many telocytes with long telopodes (T), several eosinophils with its characteristic cytoplasmic granules, some apoptotic ESCs (AS), fibroblasts (F) and collagen fibers (CF). (**B**) NETA treated group showing the endometrium contained many telocytes with long telopodes (T), several eosinophils with decreased cytoplasmic granules, ESCs (SC), fibroblasts (F) and collagen fibers (CF). (**C**) Control group showing ovoid eosinophilic cytoplasmic granules formed of clear large central crystalloid body surrounded by a less electron-dense matrix (arrowhead). (**D**) NETA treated group showing the eosinophilic cytoplasmic granules formed of ill-clear small central crystalloid body surrounded by a less electron-dense matrix (arrowhead). Some granules contained no central crystalloid body (arrow). Scale bar A–D = 2 microns.

Transmission electron microscopy of the present work explored that the endometrium in control group contained many telocytes with long telopodes, several eosinophils with their characteristic cytoplasmic granules, some apoptotic ESCs, fibroblasts and collagen fibers (Fig. 8A). However the endometrium in NETA treated group contained many telocytes with long telopodes, several eosinophils with decreased cytoplasmic granules, ESCs, fibroblasts and collagen fibers (Fig. 8B). In control group the ovoid eosinophilic cytoplasmic granules were formed of clear large central crystalloid body surrounded by a less electron-dense matrix (Fig. 8C). While in NETA treated group the eosinophilic cytoplasmic granules were formed of ill-clear small central crystalloid body surrounded by a less electron-dense matrix and some granules contained no central crystalloid body (Fig. 8D).

**Fig. 9:**
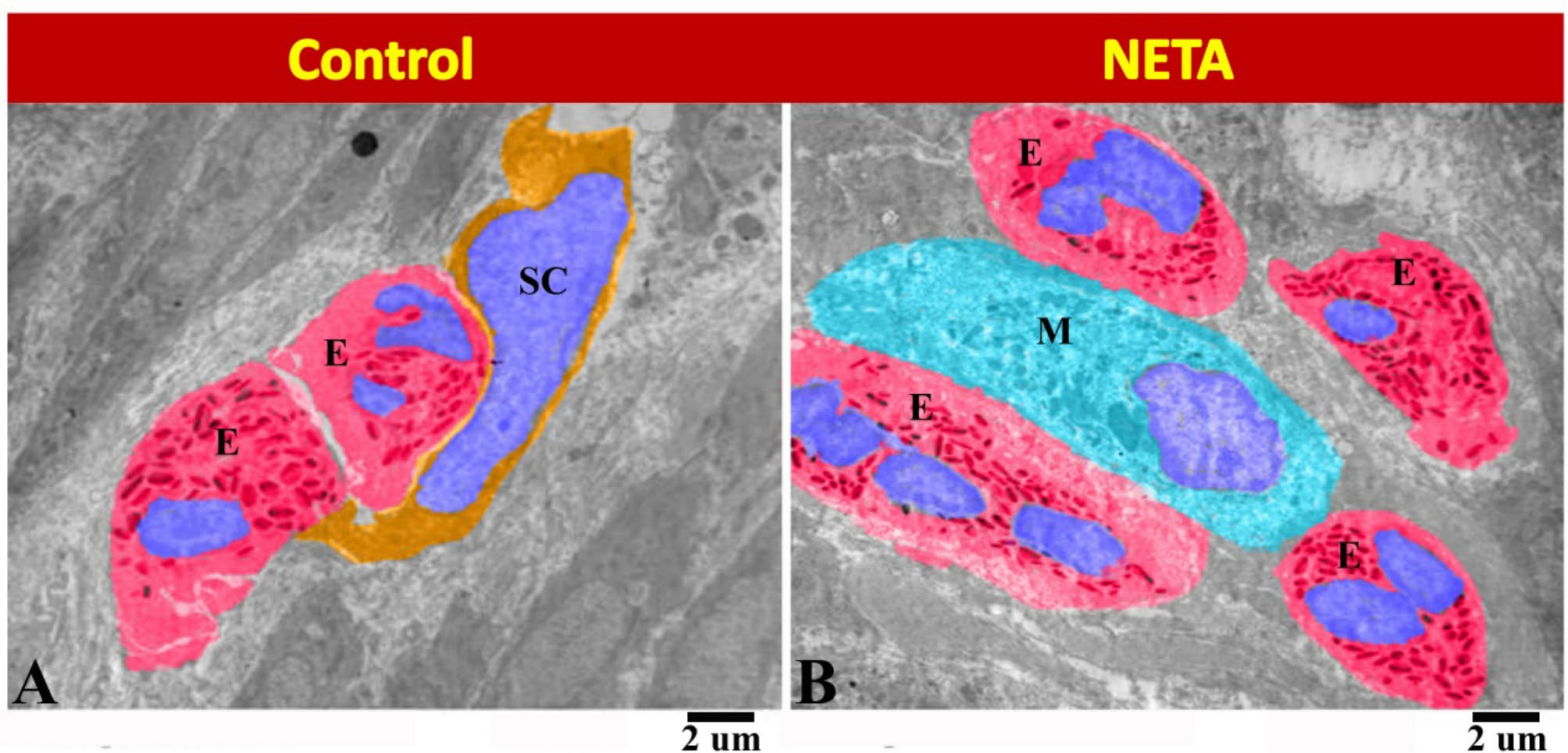
Transmission electron micrograph in the rat endometrium showing the effect of NETA on the ESCs. (**A**) Control group showing the close contact between the ESCs (SC) and eosinophils (E). (**B**) NETA treated group showing the macrophage (M) in close contact with many eosinophils (E). Scale bar A & B = 2 microns.

We found that in control group there was close contact between the ESCs and eosinophils (Fig. 9A). Whereas in NETA treated group we observed that the macrophage was in close contact with many eosinophils (Fig. 9B).

**Fig. 10:**
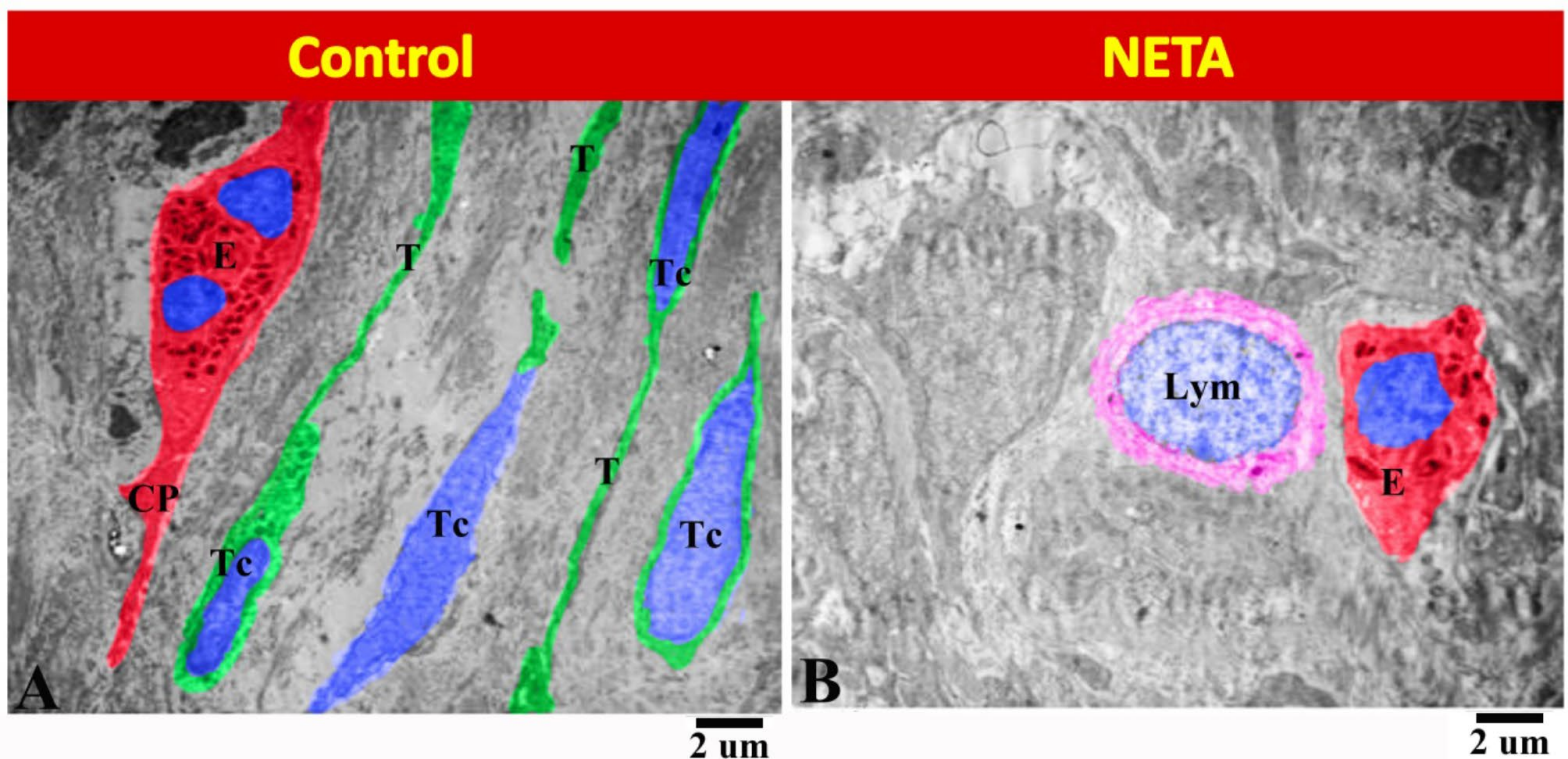
Transmission electron micrograph in the rat endometrium showing the effect of NETA on the ESCs. (**A**) Control group showing many telocytes (TC) with its characteristic elongated euchromatic nucleus and long telopodes (T) in close relation to eosinophil (E) with elongated cytoplasmic process (CP). (**B**) NETA treated group showing the lymphocyte (Lym) in close relation with eosinophil (E). Scale bar A & B = 2 microns.

The present study showed that the endometrium of the control group contained many telocytes with its characteristic elongated euchromatic nucleus and long telopodes. These telocytes were in close relation to eosinophil which had elongated cytoplasmic processes (Fig. 10A). While in NETA treated group the lymphocyte was in close relation with eosinophil (Fig. 10B).

## Discussion

Nonhuman primates and other species, such as rodents or rabbits, play crucial roles in preclinical dosing optimization, safety pharmacology, and contraceptive target discovery as well as understanding of the contraceptive mechanism of action, and evaluation of contraceptive efficacy. Despite the fact that there are interspecies variations in follicular, luteal, and endometrial dynamics, preclinical modeling advances the development of contraceptives and, in the end, increases patient safety and contraceptive choice worldwide^22^.

Contraceptives have beneficial uses in humans as well as domestic and non-domestic animals. In both human and animal models (such as rats and dogs), today’s oral contraceptives offer a high degree of effectiveness, a low incidence of nuisance side effects, and a low incidence of major adverse effects^23^. Many oral contraceptive were used in USA and Europe to control wildlife mammals and birds such as bears, rodents and wild dogs, cats, pigs and pigeons which had an economic and or health importance^4^. A potentially effective management option for captive exotic animals as well as free-roaming animals is reversible fertility control^24^. Progestin contraceptives are utilized as a financially viable method of managing feral cats as well as reversible reproductive control in genetically valuable wild felids and permanent reproductive control in generic wild felids^25^.

It has been demonstrated that norethisterone acetate, administered for four weeks beginning in prooestrus, may regulate oestrus in Greyhound bitches, preventing its symptoms and having an impact on both their fertility and racing performance^26–28^. The race results of female Greyhounds are negatively impacted by norethisterone acetate^29^. Progesterone medications was a dose and duration dependent; it work best when given to cats and dogs 7 to 15 days before estrus, and any alterations to an animal’s reproductive system can be reversed. When contraceptives are prescribed during estrus, there is a higher chance that cats and dogs will develop reproductive system pathologies like metritis, pyometra, polycystic ovaries, endometrial hyperplasia, and mixed diseases (pyometra and polycystic ovaries). While most animals’ reproductive systems develop irreversible disorders when they use drugs continuously^6^.

It is possible to synchronize mares’ estrus with synthetic progestin. Breeders who want to shorten the breeding season or who use artificial insemination and want to maximize the use of the stallion may find this interesting^11^.

At the commercial level, hormonal regulation of reproduction in small ruminants (goats and sheep) can be utilized to control the timing of oestrus (synchronization), which in turn controls the timing of kidding and lambing, as well as the weaning of young animals for slaughter. Additionally, it permits the more economical use of labor and animal facilities. Oestrus synchronization and artificial insemination can also be used to facilitate multiple ovulation and embryo transfer programs^9^. It was found that the development of fixed time artificial insemination and embryo transfer programs in dromedary she-camel depends on the synchronization of follicular waves^10^. Using a progesterone-releasing intravaginal device (PRIDΔ) is efficient to synchronize follicular wave with good pregnancy rate in camel during breeding season^30^.

Effect of oral administration of NETA on immune cells of rat uterus revealed that there was abundant infiltration of polymorph nuclear leucocytes (neutrophils and eosinophils) in superficial and deep layer of endometrium and in myometrium in NETA treated group compared to control. Our findings showed that the endometrial stroma in control group contained fibroblasts, lymphocytes, macrophages and few decidual cells. But in NETA treated group the endometrial stroma contained fibroblasts, lymphocytes, macrophages and numerous decidual cells. Oral contraceptives are associated with edematous stroma with decidual reaction and stromal granulocytes. The morphologic endometrial changes differ according to the progestin type, dose, duration, the potency, and the host receptor status and may also vary depending on whether or not estrogen is used^31,32^. The use of progestin-only contraceptive result in atrophy and marked vascular changes in uterus^32^. Assessment of estrus cyclicity in progestin-only treated rats showed a dose-response relationship in the shift to a larger number of acyclic rats and prolonged diestrus^33^. The use of Mesigyna contraceptives which contained norethisterone enanthate and estradiol valerate showed stromal hypercellularity and Polymorphonuclear cellular infiltration in both endometrium and myometrium^34^.

Decidualization is the morphological, hormonal, biochemical, and immunological preparation of the endometrium to form the decidual lining into which the blastocyst implants. The optimal decidualization of the endometrium is the key factor of pregnancy success^35,36^. Decidualization in humans begins during the latter half of each menstrual cycle independent of the conceptus^37^. Whereas, decidualization in rat only occurs during a specific period of time during pregnancy and pseudopregnancy or in ovariectomized animals when the uterus sensitized by an appropriate hormone regime^38^. The rat has hemochorial placentation with deep intrauterine trophoblast cell invasion^39^.

This process includes the decidual reaction of the ESCs, the infiltration of decidual leukocytes, glandular changes, and vascular changes to maternal arteries. The sum of these changes results in the formation of the decidua. The decidual reaction is the dramatic morphological and functional changes to ESCs. It is one of the most critical and remarkable events that occurs within the endometrium. It refers to the genetic reprogramming and terminal differentiation of elongated, fibroblast-like mesenchymal cells; ESCs in the uterine stroma to rounded, secretory, epithelioid‐like cells; decidual stromal fibroblast cells (DSCs) during the menstrual cycle in human and pregnancy in all mammals in response to elevated progesterone levels^37^. This reprogramming includes down-regulation of genes involved in the pro-inflammatory response, increase expression of genes that promote angiogenesis, foster immune tolerance, and facilitate tissue invasion. DSC is the key element in the decidual transformation. It secretes hormones, growth factors and many cytokines and the major secretory products include prolactin and insulin-like growth factor binding protein-1 (IGFBP-1), which are used as markers of decidualization^37,40–42^. Endometrial stem/progenitor cells play a role in regeneration of uterine tissue^43^.

Using immunohistochemistry we reported presence of moderate PR immunostaining immune cells in control group whereas we observed abundant strong PR immunostaining immune cells in NETA treated group. Many functions of different immune cells depend upon steroid hormone receptors. Decidualization involves the recruitment of leukocytes namely; polymorph nuclear leukocytes (neutrophils and eosinophils), mononuclear leukocytes as specialized uterine natural killer (uNK) cells, T-lymphocytes, B-lymphocytes, mast cells, macrophage and dendritic cells^41,44,45^. Interestingly, the uterine immune cells are adapted to their functions and are distinct from their peripheral pendants. They recognize foreign antigens but induce active tolerogenic responses^45–47^.

It is reported that, the migration of neutrophils from the blood vessels into the endometrial stroma occur during the time of implantation and decidualization. It is stated that sensitization for implantation is accompanied by infiltration of neutrophil polymorphonuclear leucocytes. Neutrophil provide the first line of defense against any foreign body^48^.

The present work explored that the endometrium in control group contained several eosinophils with its characteristic eosinophilic cytoplasmic granules. However the endometrium in NETA treated group contained several eosinophils with decreased cytoplasmic granules. Eosinophilic leukocytes are involving in parasitic infections and allergic reaction and are increase in number in uterus when estrogen level is elevated during estrus cycles. It was also found that ovarian estrogen negatively regulates uterine eosinophil distribution during peri-implantation period^49^. Their specific granules are large, eosinophilic, refractile, ovoid in shape, and contain a dark crystalloid body. These crystalloid bodies are effective against parasites and responsible for the refractivity of the granules in the light microscope. They contain an arginine-rich protein called major basic protein (MBP), which accounts for the intense acidophilia of the granules. The rest of the granule contains other anti-parasitic substances and histaminase^50–52^. The cytoplasm of eosinophils also contains lysosomes (azurophilic granules). Eosinophils were seen to emigrate into the uterine stroma from the blood vessels just prior to, during estrus, and 1 day postestrus. Eosinophilic leukocytes underwent degranulation to release their granules into the extracellular spaces and both whole eosinophils and individual granules from lysed cells were ingested by resident macrophages^53^. It has been hypothesized that eosinophils are cellular targets of the uteroplacental heparin-binding cytokine decidual/trophoblast prolactin-related protein. This cytokine/hormone regulates decidual cell activities needed for the initiation and maintenance of pregnancy^54^.

Estrous in rat is characterized by endometrial epithelial and glandular degeneration and necrosis which accompanied by polymorph leukocyte infiltration; especially neutrophils and eosinophils^55,56^.

uNK cells are not mainly act as cytotoxic but produce cytokines, growth factors, and chemokines during decidualization, receptivity, and implantation^45,57^.

Uterine macrophages (uMϕs) are professional and very plastic phagocytic cells that present around the rat uterine lumen. uMϕs are antigen presenting and cytokine producing cells that keeping immune homeostasis. The onset of gestation is characterized by inflammation in the context of invasion and tissue rearrangement. At the time of implantation, macrophages act as pro-inflammatory mediator cells which soon develop into tissue-remodeling macrophages. Moreover, they support trophoblast invasion and spiral artery remodeling by the secretion and regulation of the activity of matrix metalloproteinases (MMPs)^45,58^.

It has been reported that macrophages are crucial for the effective clearance of cellular debris and apoptotic cells (by efferocytosis) during uterine tissue remodeling and the invasion of the developing embryo during pregnancy to maintain tissue homeostasis^47,59^. Mϕs contribute to tissue remodeling by their destructive capacity mainly through the production of proteases and intracellular degradation in phagolysosomes. In addition to the parallel capacity of reconstructing tissues and promoting angiogenesis via the release of growth factors such as fibroblast growth factor (FGF) and vascular endothelial growth factor (VEGF), and via the production of matrix components^60^.

Accumulating evidence indicates that uterine cell apoptosis, autophagy and proliferation patterns are under the regulation of ovarian hormones. They are highly ordered cell-specific processes that play an essential role in maintaining the estrous cycle and pregnancy-associated uterine remodeling. They can result in the infiltration of immune cells, which plays an pivotal role in the endometrium shedding, tissue repair and prevention of infections during sexual cycle and pregnancy^61–65^.

Dendritic cells (DC) are antigen presenting cells, interact with T cells, NK cells, and macrophages and thus regulate the more abundant cells at the fetomaternal interface. Thereby, they support the decidualization and implantation process and pregnancy success^45,66^.

T helper and cytotoxic lymphocytes are present during decidualization and at the fetomaternal interface. They are detrimental to fetal tolerance and when activated help to create a tolerogenic environment toward the semi-allogeneic fetus, and also support the remodeling processes. They are cytokines producing cells. Additionally, several studies provided an evidence between diminished regulatory T cells frequency or disturbed activity and pregnancy complications^45,47,67,68^.

B cells are present in the decidua as they participate in the defense against infection and secret anti-inflammatory cytokines supporting the tolerogenic niche. Moreover, B cells are able to secret protective antibodies against paternal antigens to prevent fetal rejection^45,69^.

Our results showed that the presence of sub-epithelial lymphocytes and intraepithelial lymphocytes, eosinophils and macrophages indicated the crucial role of theses immune cells in epithelial and glandular remodeling during different stages of estrous cycle of control group and during degenerative changes induced by NETA. Disturbance in immune cells (types, number and functions) may leads to sexual cycle and pregnancy disturbances.

Our results revealed that tryptase positive mast cells (MCs) were present around blood vessel in the myometrium in both experimental groups. Where resting mast cell with its characteristic metachromatic granules were observed in the control group. While NETA treated group showed activated mast cell with somewhat few metachromatic granules. Uterine Mast Cells (uMCs) are predominant in the reproductive tract of rodents and humans and subjected to the hormonal influence of progesterone and estrogen as they express theses hormone receptors. It was found that estradiol augments their secretion, while progesterone inhibits histamine secretion. This explain the reduced symptoms of certain inflammatory conditions during pregnancy^70^.

The number of uMCs oscillate within the cycle, being the highest at the receptive phase, and increasing during pregnancy^47,70,71^. Based on their tissue location, murine MCs are classified as either mucosal or connective tissue-type MCs^72^. The number of MCs within the endometrium is low and predominantly localized to the basal layer^73^. They are present in connective tissue in close proximity to blood vessels, lymphatic vessels and nerves. MC is store cytokines, amines (mainly histamine), and proteases and other bioactive mediators in granules in their cytoplasm.

Activation of MC occurs by binding of immunoglobulin E (IgE) to its receptors in the surface of MCs, degranulation takes place releasing various mediators into the extracellular space^74,75^. Mast cells are involved in allergic reactions and have a unique capacity to neutralize/degrade toxic proteins, where they secrete numerous vasoactive, inflammatory and nociceptive mediators in response to immunoglobulin E (IgE) and antigen^70^. MCs synthesis and release numerous biologically active substances, some of which are pre-formed and stored in their granules for rapid release (histamine, TNF-α, heparin, lysosomal hydrolases, and proteases)^60^. Decidualization of ESCs promotes stem cell factor expression which is essential for mast cells recruitment and production of leukemia inhibitory factor that leads to successful pregnancy^76,77^.

uMCs are necessary during receptivity, implantation and pregnancy establishment as they secret histamine and other mediators. MCs promoted normal implantation, induced optimal spiral artery remodeling and favored the expression of MC proteases, transforming growth factor-*β* and connective tissue growth factor. uMCs contributed to trophoblast survival, placentation and fetal growth through secretion of the glycan-binding protein galectin-1. At later pregnancy stages, they were proposed to have rather a negative effect, as their exacerbated activation is associated with pre-term delivery^78^. It was observed that there is a dramatic increase in the number of mast cells during abortion as decidual mast cells may play an important role in the onset of abortion, due to the production of cytokines, such as tumor necrosis factor-alpha^79^. It is well-known that MCs induce myometrium contractions that are important for the induction of birth^47^.

The process of decidualization is also characterized by secretory transformation of the uterine glands and vascular changes^40–42,77^. Uterine glands and their secretions are suggested to be essential for blastocyst implantation, uterine receptivity and decidualization in the uterus of rodents and humans. One factor solely expressed by uterine glands in mice is leukemia inhibitory factor (LIF) which play a role in these processes^80,81^.

Our findings showed that the endometrium in both experimental groups contained many telocytes (TCs) with its characteristic euchromatic nucleus and long telopodes and some of them in control group showed signs of differentiated into eosinophil as they still contained telopodes. These TCs were in close relation to ESCs, fibroblasts, and immune cells (eosinophils, macrophages, and lymphocytes). We elucidated that TCs may be the key coordinator cell in endometrial stroma. As they have the ability to communicate with different epithelial and immune cells with its long telopodes and can be differentiated into different cells especially eosinophil and decidual cells. TCs might participate in cell signaling, angiogenesis, organ regeneration and repair, apoptosis, regulation of hormone-dependent processes, immune surveillance, micro environmental maintenance, and the nursing of stem cells^82–87^. Evidently, the structural-functional communication of these cells is orchestrated by complex cellular mechanisms (e.g., cell-cell contact interactions) and extracellular (paracrine, endocrine) signaling systems^83^. TCs are involved in intercellular communication by direct homo- and heterocellular junctions or by extracellular vesicle release^85^.

TCs exhibited stronger immunoreactivity for progesterone and estrogen alpha receptors and hormonal alterations during the different reproductive state impact the morphology and secretory behavior of TCs^20,88^.

Herein we found that the myometrium of NETA treated group contained many telocytes with vesicular cytoplasm compared to control. Telocytes in myometrium have different functions they may be act as stem cells for degenerative and destroyed smooth muscle cells, help in myometrium contraction or have signaling functions^82,84,85^. We proposed that TCs in uterus may be implications in many important physiological and pathological processes which should not be overlooked. They have potential roles in morphogenesis and maintenance of the normal three-dimensional architecture of tissues, repair of damaged tissue, in controlling of the stem cell microenvironment, as having anti-inflammatory and cancer-suppressing properties^89,90^.

The differences in telocytes morphology, distributions and relations to other stromal, epithelial and immune cells after oral administration of NETA may indicate that NETA may exert its contraceptive effect by direct or indirect effect on the tissue coordinator telocytes.

TCs have been found in a large variety of organs: heart, digestive tract and glands, respiratory system, urinary system, female reproductive system (uterus, Fallopian tube, placenta, mammary gland, vasculature, serous membranes, skeletal muscle, meninges and choroid plexus, neuromuscular spindles, fascia lata, skin, eye, prostate and bone marrow. Likewise, TCs are widely distributed in the female reproductive system, emphasizing their involvement in physiological and pathophysiological processes^82,87^.

Immunoexpression of PR in rat uterus after oral administration of NETA showed negative PR immunostaining in the surface epithelium and glandular epithelium of degenerated uterine glands and moderate PR immunostaining in the underlying ESCs and glandular epithelium of healthy uterine glands. Also it showed strong PR immunostaining in the inner circular and outer longitudinal smooth muscle fibers and negative PR immunostaining in the endothelium of the blood vessels in the myometrium. Similar results were obtained by^34^ when used Mesigyna in albino rats. Progesterone hormone exerts its effect on the uterus through binding to progesterone receptors (PRs). PRs are nuclear proteins which present in epithelial, stromal and smooth muscle cells of the uterus. Estrogen regulates synthesis of progesterone receptors, so their presence is a good indicator for endometrial hormonal dependence. The levels of these receptors reach its peck during pre-ovulatory and immediate post-ovulatory periods; however it decreases sharply after ovulation^20,34^. The endometrial cells are a sensitive target for steroid sex hormones. Oral contraceptives exert a predominant progestational effect as stromal decidualization with granulocytes infiltration^91^.

We hypothesize that NETA can modulate these immune cells in many ways. First, NETA could cause increase in endometrial necrosis which lead to immune cells attraction. Second, NETA exert a strong progestogenic effect. Third, NETA may have a suppressive effect on hypothalamic–pituitary–gonadal axis and lower estrogen levels.

## Conclusion

Collectively, our results indicated that the progesterone and other progestogenic substance as NETA regulate endometrial decidualization during peri-implantation period and provide an insight into a role of uterine immune system and telocytes in control of conception, embryo implantation, and pregnancy. Inadequacies in these key processes negatively impact pregnancy. We hypothesize that the primary routes of pregnancy control by NETA are the decidual (pregnancy-like) effects or improper decidualization which prevent fertilization and implantation respectively. Additionally, NETA can be used in the control the estrous cycle in wildlife and pets and synchronization of the estrous cycle in domestic animals which have health and economic importance respectively.

## Data Availability

The datasets used and/or analysed during the current study are available from the corresponding author on reasonable request.
